# A Novel Method for Soil Organic Matter Determination by Using an Artificial Olfactory System

**DOI:** 10.3390/s19153417

**Published:** 2019-08-04

**Authors:** Longtu Zhu, Honglei Jia, Yibing Chen, Qi Wang, Mingwei Li, Dongyan Huang, Yunlong Bai

**Affiliations:** 1 Key Laboratory of Bionic Engineering, Ministry of Education, Jilin University, Changchun 130022, China; 2 College of Biological and Agricultural Engineering, Jilin University, Changchun 130022, China; 3 Jilin Province Soil and Fertilizer Station, Changchun 130031, China; 4 College of Information, Jilin Agricultural University, Changchun 130118, China

**Keywords:** artificial olfactory system, soil organic matter, gas sensor array, prediction methods, regression algorithms

## Abstract

Soil organic matter (SOM) is a major indicator of soil fertility and nutrients. In this study, a soil organic matter measuring method based on an artificial olfactory system (AOS) was designed. An array composed of 10 identical gas sensors controlled at different temperatures was used to collect soil gases. From the response curve of each sensor, four features were extracted (maximum value, mean differential coefficient value, response area value, and the transient value at the 20th second). Then, soil organic matter regression prediction models were built based on back-propagation neural network (BPNN), support vector regression (SVR), and partial least squares regression (PLSR). The prediction performance of each model was evaluated using the coefficient of determination (R^2^), root-mean-square error (RMSE), and the ratio of performance to deviation (RPD). It was found that the R^2^ values between prediction (from BPNN, SVR, and PLSR) and observation were 0.880, 0.895, and 0.808. RMSEs were 14.916, 14.094, and 18.890, and RPDs were 2.837, 3.003, and 2.240, respectively. SVR had higher prediction ability than BPNN and PLSR and can be used to accurately predict organic matter contents. Thus, our findings offer brand new methods for predicting SOM.

## 1. Introduction

Soil organic matter (SOM) is defined as the sum total of all organic carbon-containing substances in the soil, which consists of the plant and animal residues at various stages of decomposition, cells and tissues of soil organisms, and well-decomposed substances [1,2]. SOM is composed of elements and compounds; the main elements include C, H, O and N, accounting for 52%–58%, 34%–39%, 3.3%–4.8%, and 3.7%–4.1%, respectively, followed by P and S, while compounds include sugars, organic acids, aldehydes, alcohols, ketones, fibers, hemicellulose, lignin, nitrogen-containing compounds, fats, waxes, resins, and tannins [3]. SOM is a key indicator of soil fertility and nutrients [4,5]. Though organic matter accounts for less than 5% of soil mass [6], it is a major energy source for soil microbes and a key nutrition source (e.g., nitrogen, phosphorus, sulfur) for crops [7]. Organic matter improves the soil physical, chemical, and biological properties of soil through different functions and can enhance soil porosity and water-conserving ability [8]. A decrease in SOM content usually implies a decline of soil quality [7]. Understanding the dynamic changes of SOM is a basic requirement for managing agricultural production and realizing precision agriculture and sustainable agricultural development [9,10,11]. Therefore, it is important to predict SOM content.

Near-infrared spectroscopy (NIRS) is fast, efficient, nondestructive, and suitable for online analysis [12]. It can measure soil parameters from a large number of samples in real time. NIRS is applicable to the development of precision agriculture and has attracted the attention of agriculture researchers [13,14,15]. SOM spectrometry is based on the spectral characteristics of soils and shows the reflectivity of organic matter at specific wave bands. The spectral curves of soils can be divided into several types according to the characteristics. Condit analyzed the spectral variations of 160 types of soils from 32 states in the US at 320–1000 nm and divided the spectral curves into three major types [16]. Krishnan et al. analyzed the correlation between soil spectral reflectivity and organic matter concentrations within 800–2400 nm and built multiple linear regression models at two optimal wave bands of 623.6 and 524.4 nm, which yielded a correlation coefficient of 0.873 [17]. However, the spectral measurement of SOM is susceptible to soil moisture. Liu et al. reported that when the moisture content is below a critical point, the soil spectral reflectivity is weakened with increased soil moisture, but above this critical point, it is intensified with increased soil moisture [18], and this critical point is usually larger than the field moisture capacity. When the soil moisture reaches the field moisture capacity, the spectral indication signals of soil organic matter nearly disappear [19]. Soil particle size and iron oxide also affect spectral measurement. Bowers and Hanks pointed out that spectral reflectivity increases exponentially as soil particles become finer, especially when the particle size is smaller 400 nm [20]. Stoner and Baumgardner showed that soil spectral curves under control by high iron oxide (>40 g/kg) can cover the impact of organic matter concentration on soil characteristics [21].

Soil gas, a component of soil, results from a balance between biological activity and gas transfer [22], which is affected by SOM content [23,24]. Under conditions of intensive oxygen consumption, the final products of the decomposition of organic compounds are carbon dioxide, water, nitrate, sulfate, and phosphate, which increases the consumption of oxygen and the carbon dioxide content in soil. However, anaerobic conditions promote the formation of gaseous hydrocarbons (CH_4_, C_2_H_4_, C_2_H_6_, C_3_H_8_, etc.), hydrogen sulfide, ammonia, and aldehydes [23]. As a consequence, soil with good air permeability has a similar composition of soil gas with near-Earth atmosphere, while soil with poor air permeability has a very different composition and soil gas with atmosphere. Therefore, the relationships between soil gas under anaerobic conditions and soil organic matter may be relevant in quantifying the SOM content. Compared with traditional chemical analysis and NIRS, the detection of SOM content by soil gas is completely new.

Metal oxide semiconductor (MOS) gas sensors, among the most important conductometric sensors [25], have the advantages of low cost, short response time, and versatility [26]. Currently, MOS gas sensors are sensitive enough for most applications [27]. However, poor selectivity is a perplexing problem that limits the widespread use of MOS gas sensors. To address this problem, many methods have been tried to improve the selectivity of gas sensors, which can be classified into three main types [27,28]: (i) Material science strategies (catalysts, filters, nanostructured coatings, etc.); (ii) sensor measurement strategies (monoclass or hybrid sensor arrays, static and dynamic measurements, etc.); and (iii) signal processing algorithms (pattern recognition methods, multivariate statistical analysis, sensor modeling, etc.). In this work, MOS gas sensors are used as soil gas detection elements.

However, it is difficult for a single MOS gas sensor to explain the complex correlation mechanism between SOM and soil gas. An artificial olfactory system (AOS) can circumvent this correlation mechanism and simplify SOM detection. An AOS, also called an electronic nose [29], is a bionic detection instrument inspired by the mechanism of biological olfaction that integrates modern sensors, electronics, and pattern recognition [30]. An AOS consists of two parts: A gas sensor array and pattern recognition elements [31]. The gas sensor array is like an olfactory receptor in a biological olfactory system, and upon contact with gases, it can convert chemical signals into electrical signals. The pattern recognition elements function like the brain of an organism and can judge, analyze, and identify electrical signals. The working principle of the AOS is that the volatile compounds in the sample contribute to the output of the sensor array as a whole rather than individuating them. The output is regarded as a unique pattern or “fingerprint” of sample gas, and different patterns can be identified by using multivariate statistical techniques and neural networks [32]. Artificial olfactory technology provides a simple and nondestructive alternative method for sample gas detection, without the need to explore the complex internal mechanisms between sample gas and sample. Thus, it is expected that the AOS can be used to detect soil organic matter, which eliminates the effects of soil texture and particle size on the detection results. AOS-based detection methods can function rapidly and simply without any complex pretreatment and are capable of online monitoring. This technique has been widely used on foods, medicines, beverages, and the environment [33,34,35,36,37,38]. Although there are some reports on artificial olfactory techniques in soil quality assessment [39,40,41,42,43,44], such as soil moisture distinction, soil microbial activity measurement, soil contamination detection, soil type identification, etc., to our knowledge, there are no papers on detecting soil organic matter by using AOS.

Given that the gases emitted from soil organic matter in anaerobic conditions contain diverse volatile organic compounds (VOCs), in this case, 10 VOC-sensitive MOS gas sensors are used as a sensor array for the AOS. As mentioned above, sensor array and pattern recognition methods can improve the selectivity, and AOS includes both of them, thus improving the selectivity of MOS gas sensors. Moreover, research has shown that temperature modulation of MOS sensors also improves selectivity [26,27]. This is because the response of the sensor depends on its working temperature, and there are usually two temperature control modes: Isothermal modulation and periodic thermal modulation [25]. Isothermal modulation only needs to provide a constant heating voltage to each sensor in the array, which has the advantages of simple operation and easy implementation. In this study, isothermal modulation is adopted, and the working temperature of each MOS gas sensor is set at equal intervals within the heating voltage range of the sensor. The aims of this study are to (1) discuss an AOS device based on a gas sensor array controlled by different temperatures and use it to predict soil organic matter; and (2) evaluate the ability of three calibrated algorithms to predict soil organic matter content: Back-propagation neural network (BPNN), support vector machine (SVM), and partial least-squares regression (PLSR).

## 2. Materials and Methods

### 2.1. Study Area and Soil Sampling

**Figure 1:**
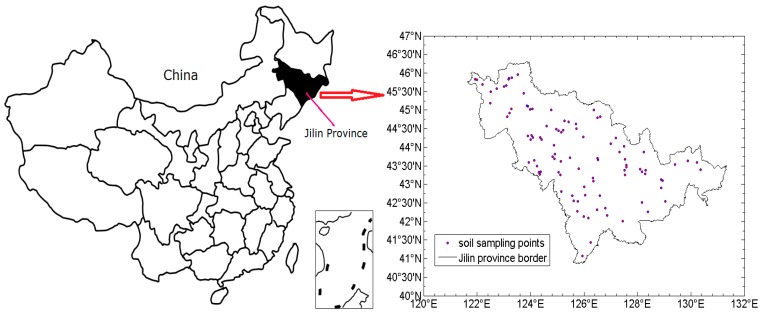
The study area and sampling sites.

The study area (40°50′ N, 121°38′ E–46°19′ N, 131°19′ E; Figure 1) is located in Jilin Province, a large part of which is in the Northeast China Plain, the largest plain in China, and covers an area of about 187,400 km^2^. It lies in a temperate continental monsoon climate zone with an annual average air temperature of 5.1 °C. The physiognomy of Jilin Province has obvious differences, and the terrain slopes from southeast to northwest, showing characteristics of high southeast and low northwest. The main soil types in this region include dark brown soil, chernozem, planosol, herbal soil, and black soil, planted mainly with corn, soybean, and wheat. Fertilization is extremely important in these soils because of soil degradation caused by frequent tillage. Thus, research in this area can help amend the soil with optimized fertilization.

A total of 102 soil samples were collected from the study area in autumn 2018. Before sampling, impurities and floating soils were removed. The sampling depth was 0–20 cm. Within a 2 m radius of each sampling site, 11 portions of soil were collected in an S-shaped way and then well mixed as one sample. Then, 1 kg of each sample was reserved based on a quartering method. According to experimental needs, the 102 soil samples were taken back to our laboratory and naturally dried in a wind-free place at 24 °C. Then, the soils were crushed and passed through a 0.25 mm sieve. After that, each sample was divided into 2 portions for chemical measurement and artificial olfactory analysis. The samples for chemical analysis were determined for SOM content by the potassium dichromate method [45], a standard examination method for SOM determination (GB9834-88, China) [46]. The principle of this method is to digest the organic carbon in soil by using a certain amount of potassium dichromate solution under heating with an electric sand bath, then the remaining potassium dichromate after digestion is titrated with ferrous sulfate standard solution, using phenanthroline as indicator. The SOM content can be calculated according to the amount of organic carbon consumption of the potassium dichromate multiplied by the constant 1.724. In this study, the results determined by this method are called observed values. The samples for artificial olfactory analysis were stored in bags.

### 2.2. Artificial Olfactory Measurements

#### 2.2.1. Measurement Setup and Data Acquisition

**Figure 2:**
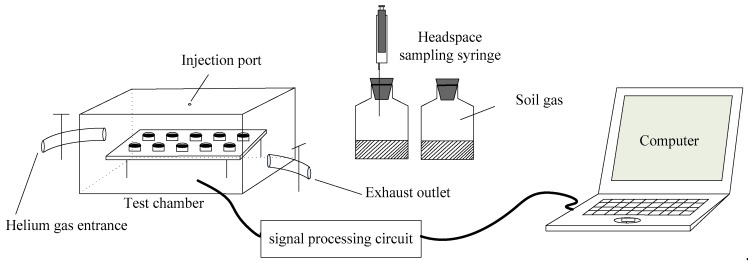
Artificial olfactory measurement setup.

To perform the experiment successfully, a measurement setup with a fully computerized system was needed. The artificial olfactory device consisted of an array of 10 MOS sensors (installed in a closed test chamber), a signal processing circuit, and a laptop computer (Figure 2). The signal processing circuit consisted of 10 temperature modulated circuits (corresponding to the 10 MOS sensors) and an STM32 microprocessor, which was used to collect and process sensor output signals. The signal processing circuit and the laptop communicated through an RS232. The sensor array was connected via an FFC soft line to the signal processing circuit.

**Figure 3:**
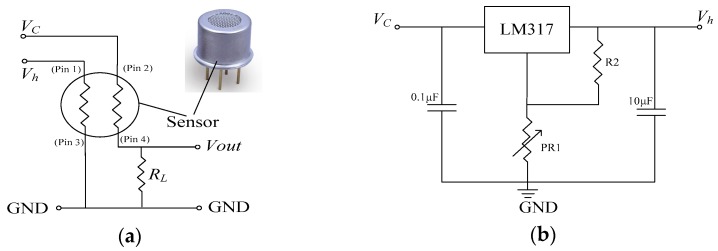
Sensor circuit: (**a**) The basic measuring circuit of sensors; (**b**) temperature modulation circuit.

A sensor array is the basis of an artificial olfactory system, and a reasonable sensor array is the key to improving the overall performance of the system. In the construction of a sensor array, each gas sensor or element should have some cross-sensitivity. The cross-sensitivity can not only reduce the requirement of sensor selectivity, but also improve the efficiency of the array. The AOS makes use of the cross-sensitivity of the sensor array to achieve system selectivity and improved measurement accuracy. In this study, the IDT SGAS707 type gas sensors, purchased from Integrated Device Technology Inc. (San Jose, CA, USA), were used to construct an array for the detection of VOCs in soil gas. The sensors use an integrated heater with highly sensitive polymer-MOx composite material designed especially for detection of VOCs [47]. The sensors have the following advantages: (i) High sensitivity to a wide range of VOCs; (ii) responds to many different organic vapors (nonspecific); (iii) small temperature and humidity effects; and (iv) long sensor life and high repeatability. Figure 3 shows the basic measuring circuit of the sensors as well as the temperature-modulated circuit.

The working of the sensor required 2 applied voltages: Circuit voltage (*V_c_*) and heater voltage (*V_h_*). *V_c_* provided the temperature modulation circuit with a working voltage and measured the output voltage *V_out_* across the load *R_L_*. *V_out_* can be calculated as follows:

(1) $$V_{out}=\frac{R_{L}}{R_{L}+R_{s}}V_{c}$$

where *R_s_* is the output resistance of the sensor and nonlinearly declines with increasing gas concentration [30]. *V_h_* is a constant heating voltage (*V_h_* ≤ 3.5 V at an ambient temperature of about 24 °C) used for working temperature control and raises the selectivity of the sensor array, and can be set through the temperature modulation circuit. To facilitate the modulation of *V_h_*, the temperature modulation circuit was designed with one LM317 3-terminal voltage regulator (Figure 3b). Through the RP1 resistance potentiometer, *V_h_* can be set to different values above 1.25 V, which is limited by the regulatory capacity of the LM317, but suitable for SGAS707 sensors. In this study, the values of the heater voltage *V_h_* of the 10 MOS gas sensors were set with a step of 0.25 V in a range from 1.25 V to 3.5 V. The corresponding temperature was measured by a PT1000 platinum resistance thermometer (precision class B) attached to the metal shell of the MOS gas sensor, listed in Table 1.

The 102 soil samples for artificial olfactory analysis (each 80 g) were sprayed with distilled water and naturally wind-dried, which ensured a relative humidity of 65%. After that, each soil sample was put into a 250 mL gas-collecting vial and sealed with a rubber plug to create anaerobic conditions. Before the measurement, the gas-collecting vials were stored in a dark room ventilated with air at a temperature of 24 ± 1 °C for at least 24 h. This meant that the saturated soil gas could be maintained in the vial headspace. Since the measurements were taken one after the other, in order to ensure that all samples had the same sealing time, the soil gas in the vials was transferred to 200 mL foil gas sampling bags by 20 mL syringes for temporary storage. At the beginning of the measurement, 20 mL of soil gas was extracted from the sampling bag and injected into the closed test chamber through the injection port. To avoid gas leakage from the chamber, the injection port was sealed with sealing cement. When *V_h_* and *V_c_* were used in different sensors, the outputs from the 10 sensors were acquired simultaneously by the 10-bit, 10-channel A/D converters inside the STM32 microprocessor and recorded on the hard disk of the computer. After each measurement, the test chamber was rinsed with inert helium gas, and after the output voltages from the sensors had stabilized, the next measurement was started. It is worth mentioning that the measurements should be carried out under constant experimental conditions. The typical values used in the experiments of this study were as follows: Test chamber volume, 140 mL; soil gas volume, 20 mL; ambient temperature, 24 °C.

**Figure 4:**
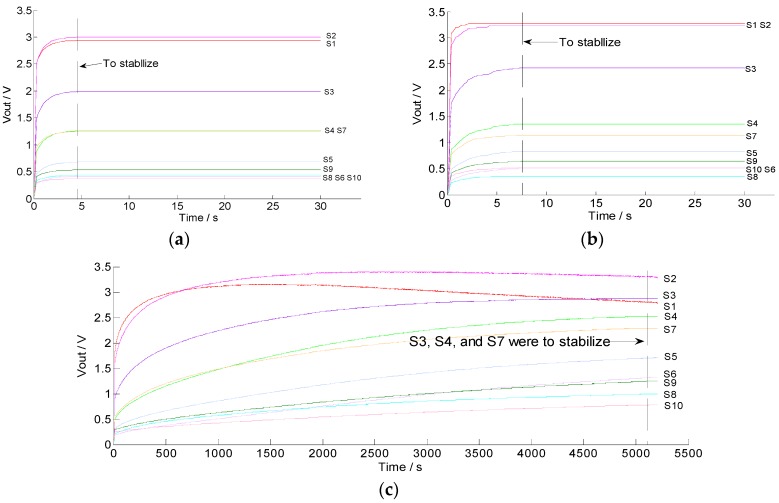
Response curves of the sensors: (**a**) Helium; (**b**) air; (**c**) soil gas.

Generally, a high sampling frequency will better reflect the response of the sensors, but will increase the difficulty of data processing in the later stage; on the other hand, a low sampling frequency will cause the loss of key data. In our study, the sampling frequency of the A/D converters over the sensors was 10 Hz and the duration was 5 min. Moreover, a one-dimensional median filtering algorithm was used to eliminate the noise interference in the early stage of data processing. Figure 4 shows the response curves of the sensor array. It can be seen that the 10 Hz sampling frequency was effectively able to obtain the response change of the curves and ensure an appropriate amount of data. Under helium and air conditions, the response curves of the sensor array were quickly stabilized, but the former was faster than the latter (Figure 4a,b). This may be due to the presence of a little organic volatile gas in the air. Therefore, the helium was more suitable as the rinsing gas in this study. The measurement results show that the 10 sensors had similar but different response characteristics, indicating that all the sensors in the sensor array could work appropriately without any redundancy (Figure 4c). However, it took too long (>80 min) for all sensors in the sensor array to stabilize, which was extremely detrimental for fast measurements. To improve the detection efficiency and reduce time consumption, we only used the data from the first 5 min of each test.

#### 2.2.2. Feature Selection

The extracted features should reflect the response curves of the sensors. The output from the sensor array was a time series of output signals from the 10 sensors. Features can be extracted by many methods, including extracting transient values, steady values, or both. However, the transient response usually reflects the different dynamic behaviors of a sensor under contact with different gases (or odors) and may contain more information than the steady signals [48]. Moreover, the use of transient response reduces the time required for data acquisition. Commonly used transient feature extraction values include (i) maximum or minimum value (Vmax or Vmin), (ii) mean differential coefficient value (MDCV), (iii) response area value (RAV), (iv) time to reach the maximum voltage (Tm), and (v) transient time value (Vf). For example, Fu et al. used Vmax, Tm, Vf, and RAV as the features to construct characteristic vectors of the VOC response curves and achieved a good classification and recognition effect [49]. In this study, 4 features (Vmax, MDCV, RAV, and transient value at the 20th second (Vt)) were extracted from the response curves to build the eigenvectors. MDCV and RAV can be defined as follows:

(2) $$\mathrm{MDCV}=\frac{1}{N-1}\sum_{i}^{N-1}\frac{D_{i+1}-D_{i}}{\Delta t}$$

(3) $$\mathrm{RAV}=\sum_{i=1}^{N}D_{i}\cdot \Delta t$$

where *N* is the number of pieces of data measured by one sensor (3000 in this study); *D_i_* is the *i*-th sampling data; and △*t* is the time interval between 2 adjacent samples (here, 0.1 s).

To eliminate the effects of orders of magnitude and gas concentrations on the predicted results, we normalized the extracted features as follows:

(4) $$X_{new}(i)=\frac{X(i)-u(x)}{S(x)}$$

where *X(i)* stands for Vmax, MDCV, RAV, and Vt. *u(x)* and *S(x)* are the mean and variance of features from the 10 sensors and can be calculated as follows:

(5) $$u(x)=\frac{1}{10}\sum_{i=1}^{10}X(i)$$

(6) $$S(x)=\sqrt{\frac{1}{9}\sum_{i=1}^{10}{\left(X(i)-u(x)\right)}^{2}}$$

According to the discussion above, each soil sample contained 40 features (4 features × 10 sensors), for a total 102 × 40 olfactory feature space.

### 2.3. Regression Calibration Models

The term “calibration” refers to 2 processes. First, it establishes the relationship between indicative measurement and standard (reference) measurement, i.e., estimating the parameters of a calibration model (or function); second, it uses the established calibration model to obtain measurement results from indicative measurements [50,51]. In this study, the purpose of calibration is to establish the relationship model between olfactory feature space and SOM content, so as to predict (or determine) the content of unknown soil organic matter. SOM content, which may be linearly or nonlinearly correlated with the response curves of the gas sensor array, can usually be calibrated by BPNN, SVR, and PLSR models.

#### 2.3.1. BPNN

BPNN is a multilayer forward neural network based on error reverse dissemination and is the most widely used neural network [52]. The creation of BPNN depends on the following factors: Number of input variables, number of output variables, number of hidden layers, and numbers of neurons in different layers. The numbers of input and output variables should correspond to the numbers of features extracted and the number of prediction indices, respectively. Here, 40 features were extracted from each sample, and the variable to be predicted was soil organic matter content. Thus, the numbers of input variables and output variables were 40 and 1, respectively. The Kolmogorov theory proves that a 3-layer network containing 1 hidden layer can approximate any nonlinear function [53,54,55]. Thus, the number of hidden layers was set as 1. However, the number of neurons in the hidden layer largely affects the BPNN. If this number is too small, the network cannot fully describe the relationship between the input and output variables, but if the number is too large, the learning time of the network will be prolonged, which can cause overfitting. So far, there is no precise equation to calculate the neuron number of the hidden layer, but its range can be determined by empirical formulas. The following is a common equation:

(7) $$h=\sqrt{n+p}+\alpha$$

where *h*, *n*, and *p* are the numbers of neuron nodes in the hidden layer, the input nodes, and the output nodes, respectively; and α is a real number from 1 to 10. In this case, *n* is equal to the number of input variables (40) and *p* is equal to the number of output variables (1). Thus, *h* is assigned between 6 and 16.

Root mean square error (RMSE) is a major indicator of model fitting, and a smaller RMSE indicates that the prediction is more stable. In this study, root mean square error of training set (RMSET) and coefficient of determination of training set (R^2^_T_) were used to evaluate the BPNN model and provided a basis for determining the number of neuron nodes in the hidden layer. RMSET and R^2^_T_ are defined as follows:

(8) $$\mathrm{RMSET}=\sqrt{\frac{1}{m}{\sum_{i=1}^{m}\left({\hat{y}}_{i}-y_{i}\right)}^{2}}$$

(9) $$R^{2}{}_{T}=1-{\sum_{i=1}^{m}\left({\hat{y}}_{i}-y_{i}\right)}^{2}/{\sum_{i=1}^{m}\left({\hat{y}}_{i}-\frac{1}{m}\sum_{i=1}^{m}y_{i}\right)}^{2}$$

where *m* is the sample number in the training set; *y_i_* is the observed value of the *i*-th sample in the training set; ${\hat{y}}_{i}$ is the predicted value of *y_i_*.

#### 2.3.2. SVR

SVR is based on support vector machine for solving regression problems [56,57], which is one of the most important predictive statistical models [58]. The LIBSVM toolbox offers 2 types of regression methods, ε-SVR and ν-SVR [59]. Here ε-SVR was used with the radial basis function (RBF) as the kernel function. The generalization ability of SVR is affected by 2 parameters, the punishment factor C (C > 0) and the kernel parameter σ^2^ [60]. A larger C means the model has less tolerance for errors, leading to overfitting; a smaller C means the model is prone to underfitting. An excessively large or small C will weaken the generalization ability of the model. σ^2^ is a built-in parameter of BRF and implicitly decides the distribution of the data mapped to the new feature space; a larger σ^2^ means a smaller number of support vectors, and vice versa. The number of support vectors affects the speed of training and prediction. Thus, it is necessary to adjust *C* and σ^2^. For other parameters (e.g., the insensitive loss function ε), the default values offered in LIBSVM can be used. To determine the appropriate C and σ^2^ to improve the model’s prediction ability, here we optimized the 2 parameters by 5 steps: (1) Give broad initial ranges so that C and σ^2^ are both within 2^−20^ and 2^20^; (2) based on the initial ranges, build grids with large step-by-step values; (3) with fivefold cross-validation, roughly select the optimal values through the mean square error of cross-validation (MSECV); (4) roughly select the optimal values, narrow down the grid ranges, and rebuild the grids with smaller step-by-step values; and (5) use the five-fold cross-validation again and, according to MSECV, precisely select the optimal values within the grids. If the parameter combination (C, σ^2^) causes overfitting or underfitting, the grid range can be further narrowed down, and step 5 can be repeated until the requirement of prediction is met.

MSECV is computed as follows:

(10) $$\mathrm{MSECV}={\sum_{i=1}^{k}\left({\hat{y}}_{i}-y_{i}\right)}^{2}/k$$

where *k* is the number of training set samples, *y_i_* is the observed value of the *i*-th training sample, and ${\hat{y}}_{i}$ is the predicted value of *y_i_*.

#### 2.3.3. PLSR

In this study, PLSR was used as one of the prediction models of soil organic matter. PLSR is very effective at predicting a set of dependent variables from a large set of independent variables [61]. This is a new multivariable statistical data analytical method that integrates principal component analysis (PCA) and multivariable linear analysis. When the variables are highly linearly correlated, PLSR can return a very effective prediction. To overcome the collinearity between predictors, PLSR adopts a linear combination to separate independent variables and the dependent variable, so as to extract principal component factors (PCFs, or latent variables) [62]. In PLSR, the regression model was built based on PCFs (rather than the initial training variables). Thus, appropriate determination of PCF is an effective way to fully utilize the gas sensor array information and filter noise. Moreover, a suitable number of PCFs can effectively avoid overfitting or underfitting. Here, leave-one-out cross-validation was used to determine the number of PCFs reserved in the PLSR model [63]. The effect of the PCF number on the model performance is usually assessed using root mean square error of cross-validation (RMSECV) and the Akaike information criterion (AIC) [64,65]. RMSECV and AIC are respectively defined as follows:

(11) $$\mathrm{RMSECV}=\sqrt{{\sum_{i=1}^{m}\left({\hat{y}}_{i}-y_{i}\right)}^{2}/m}$$

where *m* is the number of training samples, *y_i_* is the observed value of the *i*-th training sample, and ${\hat{y}}_{i}$ is the predicted value of *y_i_*; and

(12) AIC=N⋅log(RSS)+2p

where *N* is the sample number, *p* is the number of PCFs, and *RSS* is the sum of squared residuals.

### 2.4. Training Set and Validation Set

To train and validate the models, we divided the 102 soil samples into 2 groups at a ratio of 70%/30% by using the Kennard–Stone algorithm in the feature space matrix [66]. Thus, 71 and 31 samples were used in the training set and validation set, respectively.

### 2.5. Assessment of Models

The coefficient of determination (R^2^) is often used to assess the prediction precision of models; R^2^ close to 1 implies stronger prediction ability. The ratio of performance to deviation (RPD) can be used to further evaluate the prediction effect and precision of a model, which compensate the demerits of R^2^ in the prediction of nonlinear models. Here R^2^, RMSE, and RPD were all used. Let *n* be the sample number and *y_i_* be the observed value of the *i*-th sample; *f_i_* is the predicted value of the *i*-th sample and SD is the standard deviation of *y_i_*. For different processes, the above parameters (*n*, *y_i_*, and *f_i_*) were taken from different datasets. Training set data were used for calibration and validation set data were used for prediction. RPD and R^2^ can be defined as follows [64]:

(13) $$R^{2}=\frac{{\left(\sum_{i=1}^{n}\left(f_{i}-\frac{1}{n}\sum_{i=1}^{n}f_{i})(y_{i}-\frac{1}{n}\sum_{i=1}^{n}y_{i}\right)\right)}^{2}}{\sum_{i=1}^{n}{\left(f_{i}-\frac{1}{n}\sum_{i=1}^{n}f_{i}\right)}^{2}\sum_{i=1}^{n}{\left(y_{i}-\frac{1}{n}\sum_{i=1}^{n}y_{i}\right)}^{2}}$$

(14) $$\mathrm{RPD}=\mathrm{SD}/\mathrm{RMSE}=\sqrt{\sum_{i=1}^{n}{\left(y_{i}-\frac{1}{n}\sum_{i=1}^{n}y_{i}\right)}^{2}/{\sum_{i=1}^{n}\left(f_{i}-y_{i}\right)}^{2}}.$$

In most cases, a larger RPD means higher consistency between predicted and observed values. In different research fields, the explanations of RPD differ. RPD in the prediction of soil properties is usually divided into 3 types [67]: Type A (RPD > 2.0) with high prediction ability, type B (1.4 < RPD < 2.0) with moderate prediction ability, and type C (RPD < 1.4) with weak prediction ability. The above feature extraction, data preprocessing, and model algorithms were all finished on MATLAB (MathWorks, Natick, MA, USA). The processing of SVR was conducted with the LIBSVM toolbox (LIBSVM-3.23, C.C. Chang, C.J. Lin) [68].

## 3. Results and Discussion

### 3.1. Chemical Analysis Results of SOM Content

The statistical results of SOM content (observed values) by chemical analysis for the training and validation sets are listed in Table 2. The datasets in Table 2 show dramatic variations. In the training set, the SOM content ranged from 12.37 to 43.85 g/kg, and the coefficient of variation (CV) was 31.64%. For the validation set, the SOM content ranged from 12.19 to 48.79 g/kg, and the CV was 32.90%. These results indicate that SOM content in the study area shows a spatial variation trend. Large soil variability may be beneficial to improve the prediction ability of models [69]. The range of SOM content in the validation set fully covered the range of SOM content in the training set. Given this result, the generalization ability of models will be better presented.

### 3.2. Responses of Sensors to Soil Gas

**Figure 5:**
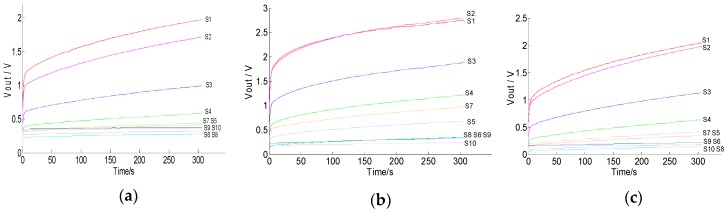
Sensor array signals of soil samples: (**a**) Soil organic matter (SOM) content 12.19 mg/kg; (**b**) SOM content 23.11 mg/kg; (**c**) SOM content 48.79 mg/kg.

The sensitivity and selectivity of the artificial olfactory device were tested by comparing different soil gas response signals. The soil gas response data were collected from three representative soils with organic matter of 12.19 mg/kg (minimum in all samples), 23.11 mg/kg (moderate in all samples), and 48.79 mg/kg (maximum in all samples). The response curves of the sensor array to soil volatiles with different organic matter contents were obtained (Figure 5). In Figure 5, the output voltages of the sensors show large differences at the same moments, which indicates that the response of each sensor to the change of gas emitted from soil is quite different, and shows the selectivity and cross-sensitivity. It is similar to the response of biological olfactory cells to odor; that is, the biological system can identify odors by generating olfactory signals and sending them to multiple olfactory cells and integrating and establishing olfactory fingerprints. The overall signal curves of soils with different organic matter have largely varying amplitudes (Figure 5a,c), indicating that the response intensity of the sensor to the gas emitted from the measured sample changed, which means that the sensor array has good sensitivity to the change of soil gas. This shows that the designed artificial olfactory device is reasonable, and the reaction time (5 min) of the interaction between soil gas and gas sensor in the test can also meet the requirements of classification and recognition.

### 3.3. Calibration and Prediction by BPNN Model

To develop the BPNN model, we used the Neural Network Toolbox in MATLAB 7.14.0.739 (R2012a) (MathWorks Inc., Natick, MA, USA). In the BPNN modeling, the activation function of hidden layer neurons was the S-shaped transfer function tansig, while in the output layer it was the linear transfer function purelin. We used the newff function to create a BP neural network with a maximum number of iterations of 1000, a learning rate of 0.01, and a target error of 0.001. After that, the created network was trained by the training set data.

**Figure 6:**
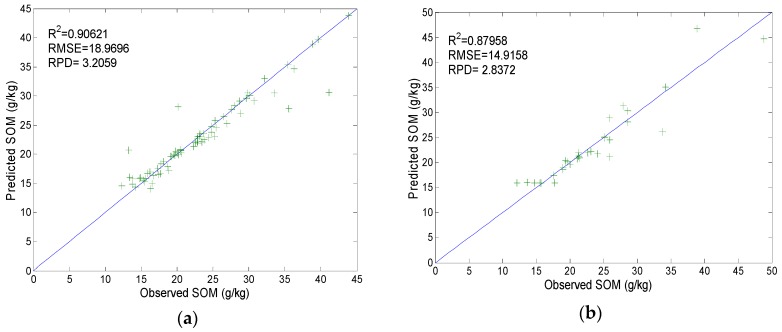
Back-propagation neural network **(**BPNN) predicted values and observed values of SOM: (**a**) Training set; (**b**) validation set.

To optimize h, within the range of h determined above, for each value of h, the training set was repeatedly tested 10 times by using the trained BPNN. Based on the running results (Table 3), the mean values of R^2^_T_ and RMSET were used as the evaluation indices. A larger mean R^2^_T_ and smaller mean RMSET suggest a better model. When h is 6, the mean R^2^_T_ maximizes to 0.793 and the mean RMSET minimizes to 21.351 (Table 3). Therefore, the calibrated BPNN is of the structure 40-6-1 (40 input variables, 6 hidden layer neurons, and 1 output variable), and its calibration results and prediction results are shown in Figure 6.

The results are R^2^ = 0.906, RMSE = 18.970, and RPD = 3.206 (calibration, Figure 6a); and R^2^ = 0.880, RMSE = 14.916, and RPD = 2.837 (prediction, Figure 6b), indicating the model has higher generalization ability when the hidden layer of the BPNN contains six neurons.

### 3.4. Calibration and Prediction by SVR Model

**Figure 7:**
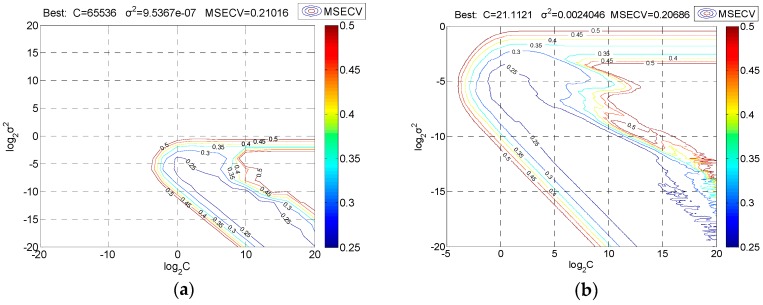
Support vector regression (SVR) parameters selection: (**a**) Contour of rough selection; (**b**) contour of precise selection. log_2_C: Logarithm of C with the bottom number 2; log_2_σ^2^: Logarithm of σ^2^ with the bottom number 2.

To improve the prediction precision of SVR, grid searching and fivefold cross-validation were performed to first roughly select and then precisely select each combination of C and σ^2^. The optimal search areas were determined by using the contour lines of MSECV plotted in Figure 7.

Figure 7 shows the results of the SVR parameters as selected. Clearly, the rough optimal values of C and σ^2^ are 65,536 and 9.5367 × 10^−7^, respectively, and the optimizing ranges of C and σ^2^ can be narrowed down to 2^−^^5^ to 2^20^ and 2^−20^ to 2^0^, respectively (Figure 7a). The exact optimal values of C and σ^2^ are 21.1121 and 0.0024046, respectively (Figure 7b).

**Figure 8:**
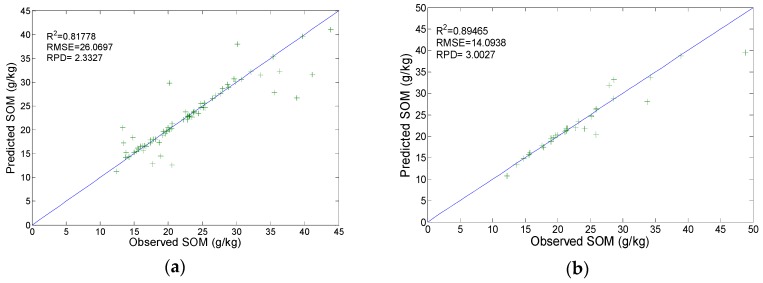
Calibration results and prediction results of SVR model: (**a**) Calibration; (**b**) prediction.

The optimized C and σ^2^ were used to construct an SVR model to calibrate the AOS. To observe the prediction of this model, we tested it using the validation set. Results show R^2^ = 0.818, RMSE = 26.0697, and RPD = 2.333 in the training set (calibration, Figure 8a); and R^2^ = 0.895 and RPD = 3.003 in the validation set (prediction, Figure 8b). This indicates that the precisely selected combination (C = 21.1121, σ^2^ = 0.0024046) has good predictive performance.

### 3.5. Calibration and Prediction by PLSR Model

**Figure 9:**
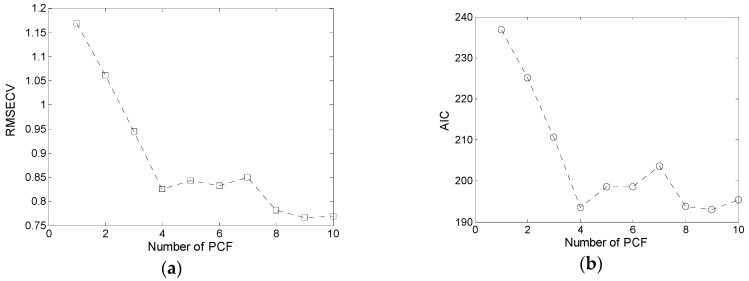
Number of principal component factors (PCFs) in partial least squares regression (PLSR): (**a**) Root mean square error of cross-validation (RMSECV); (**b**) Akaike information criterion (AIC).

The PLSR was run on the training set, and the number of optimal PCFs was determined from leave-one-out cross-validation. The RMSECV and AIC of cross-validation changed with the number of PCFs (Figure 9). The optimal number of PCFs should be selected based on a small RMSECV and AIC. Moreover, fewer PCFs can reduce the model complexity. Thus, we used four PCFs in the PLSR model.

**Figure 10:**
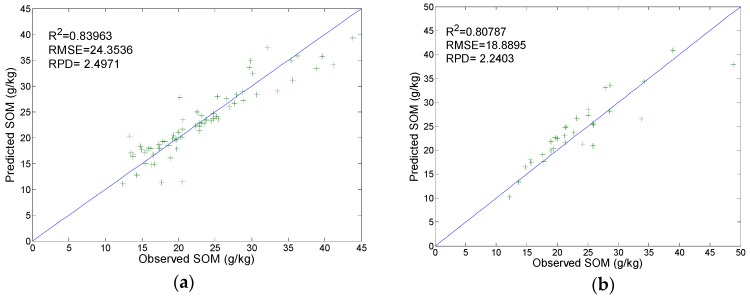
Calibration and prediction results with the PLSR model: (**a**) Calibration; (**b**) prediction.

A PLSR model was built with the PCFs as determined and was tested using the training set and validation set. The results are R^2^ = 0.840 and RPD = 2.498 (calibration, Figure 10a), and R^2^ = 0.808 and RPD = 2.240 (prediction, Figure 10b). The test results suggest the fitting effect of the PLSR model is satisfactory.

### 3.6. Model Comparison

Many regression models can achieve high prediction accuracy or explanatory power. Thus, it is necessary to select the best ones for modeling and predicting SOM. To compare the prediction performance (or generalization ability) of BPNN, SVR, and PLSR models and determine the optimal soil organic matter olfactory detection model, we used the calibrated BPNN, SVR, and PLSR models to predict the validation set. From Figure 6a, Figure 8a, and Figure 10a, it can be seen that BPNN has the best calibration effect (largest R^2^ and PRD and smallest SMSE) in the three regression calibration models of BPNN, SVR, and PLSR; among them, PLSR is the second best and SVR is the worst. However, the PRD value of the three models in the training set is greater than 2.0, indicating that the calibrations of these models are successful.

**Figure 11:**
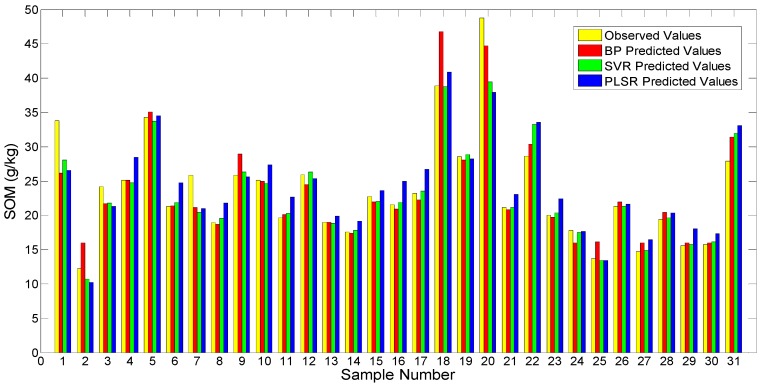
Comparison of prediction results from different models.

Calibration of the AOS was performed in the learning stage, when the olfactory feature space was associated with the already known content of SOM. After this, the learned parameters were frozen and the prediction, applying the validation set data not used in the learning stage, was carried out. Figure 6b, Figure 8b, and Figure 10b show the prediction results of these models. For more direct comparison, we plotted the prediction results of different models in Figure 11 and list relevant indices (R^2^, RMSE, RPD) in Table 4. It can be seen from Figure 11 that for samples 1, 7, 20, 22, and 31, the differences between the predicted and observed values of the three models are large. The main reason may be that the artificial olfactory measurement of one or a few samples in the training set produced errors, which could be caused by improper operation, errors in the artificial olfactory device itself, or external factors such as temperature and humidity.

According to the classification methods of RPD for soil properties, all three models are type A (RPD > 2.0) (Table 4) and all have R^2^ greater than 0.8. This indicates that all these models show high prediction ability. However, the RPDs of BPNN and SVR are much larger than that of PLSR (with a difference of more than 0.5), and the RMSEs of the two models are much smaller than that of PLSR. Furthermore, the R^2^ values of BPNN and SVR are significantly higher than that of PLSR and closer to 1. Among the models, SVR is most accurately predicted with the best RPD (3.003) and R^2^ (0.895), as well as the smallest RMSE (14.094). Therefore, the prediction performance relationship of the models is SVR > BPNN > PLSR.

It can be seen that SVR and BPNN achieve higher prediction performance than PLSR, probably because SOM is nonlinearly associated with the olfactory feature space to some extent, while SVR and BPNN are more suitable than PLSR for nonlinear regression. SVR performs better than BPNN. The main reason for this is perhaps the stronger model learning ability of SVR, since it uses a pair of optimal parameter combinations (C, σ^2^).

## 4. Conclusions

This study is one of the first attempts to indirectly predict SOM using an artificial olfactory system with various existing models. In this study, BPNN, SVR, and PLSR calibration algorithms were applied to establish the relationship model between olfactory feature space and SOM content. Results presented in Table 4 indicate that these three predictive models show high prediction ability to evaluate SOM with reasonable accuracy, with SVR showing the most accuracy, followed by BPNN and PLRS. This performance could possibly be attributed to the nonlinear correlation between SOM and olfactory feature space to some extent, and SVR’s stronger learning ability. Consequently, the SVR model may serve as a useful tool for evaluating SOM content with moderate accuracy.

The conclusions suggest that artificial olfaction is feasible for detecting soil organic matter content. The methodology can be considered robust, since the soil samples in the study area had spatial variability. These findings may offer a new basis for predicting and simplifying the measurement of soil organic matter. In the future, appropriate methods for eliminating abnormal samples should be adopted, and more calibration methods should be tested in order to reduce RMSE and increase R^2^ and RPD. Moreover, the effects of various factors (including working temperature, soil moisture, soil sealing time, etc.) on sensor selectivity/sensitivity should be studied, in order to optimize the operating parameters of the AOS.

## Figures and Tables

**Table 1:** *V_h_* of different sensors.

Sensor Number	*V_h_* (V)	Working Temperature (°C)	Sensor Number	*V_h_* (V)	Working Temperature (°C)
S1	1.25	34.4	S6	2.50	48.1
S2	1.50	36.0	S7	2.75	52.5
S3	1.75	37.8	S8	3.00	60.0
S4	2.00	40.4	S9	3.25	65.7
S5	2.25	43.0	S10	3.50	74.3

**Table 2:** Organic matter concentrations in soil samples.

Dataset	SOM (g·kg^–1^)	Max (g·kg^–1^)	Min (g·kg^–1^)	Mean (g·kg^–1^)	SD (g·kg^–1^)	CV (%)
Training set	20.51; 27.62; 33.50; 20.23; 23.11; 24.43; 28.71; 26.53; 18.88; 26.92; 14.97; 20.48; 17.69; 13.76; 17.38; 19.97; 32.13; 29.87; 28.85; 39.64; 12.37; 17.33; 14.22; 22.85; 15.49; 22.85; 25.27; 22.55; 18.13; 20.52; 25.20; 23.72; 13.44; 16.24; 15.67; 41.10; 22.31; 20.17; 13.29; 19.54; 35.55; 36.28; 43.85; 19.14; 25.42; 19.79; 13.79; 15.90; 30.71; 19.27; 23.16; 30.14; 24.76; 23.80; 27.95; 20.60; 22.88; 24.75; 23.46; 18.67; 35.38; 16.53; 15.32; 16.31; 16.74; 17.78; 22.89; 14.80; 29.65; 38.86; 19.750	43.85	12.37	22.98	7.27	31.64
Validation set	33.77; 12.19; 24.15; 25.11; 34.24; 21.32; 25.86; 18.94; 25.85; 25.10; 19.64; 25.94; 18.96; 17.58; 22.71; 21.50; 23.18; 38.92; 28.58; 48.79; 21.13; 28.62; 20.01; 17.78; 13.64; 21.28; 14.72; 19.37; 15.59; 15.71; 27.89	48.79	12.19	23.49	7.73	32.90

**Table 3:** Effects of neuron number in the hidden layer on back-propagation neural network (BPNN) performance.

Neuron Number	R^2^_T_			RMSET		
	Min	Max	Mean	Min	Max	Mean
6	0.627	0.906	0.793	15.794	26.600	21.351
7	0.382	0.810	0.678	18.841	37.911	25.515
8	0.450	0.845	0.630	18.955	31.600	26.440
9	0.280	0.824	0.690	18.190	40.136	25.424
10	0.512	0.832	0.650	17.906	36.760	27.188
11	0.503	0.804	0.716	28.880	34.671	24.384
12	0.568	0.832	0.704	18.252	30.010	24.299
13	0.391	0.867	0.726	17.611	33.872	23.202
14	0.127	0.848	0.599	16.768	55.106	32.411
15	0.561	0.812	0.681	18.927	40.192	27.247
16	0.300	0.857	0.672	17.897	37.628	25.187

**Table 4:** SOM prediction performance indices of different models.

Models	R^2^	RMSE	RPD	Category
BPNN	0.880	14.916	2.837	A
SVR	0.895	14.094	3.003	A
PLRS	0.808	18.890	2.240	A
